# Incorporation of a Hydrophilic Spacer Reduces Hepatic Uptake of HER2-Targeting Affibody–DM1 Drug Conjugates

**DOI:** 10.3390/cancers11081168

**Published:** 2019-08-14

**Authors:** Haozhong Ding, Mohamed Altai, Sara S. Rinne, Anzhelika Vorobyeva, Vladimir Tolmachev, Torbjörn Gräslund, Anna Orlova

**Affiliations:** 1 Department of Protein Science, KTH Royal Institute of Technology, Roslagstullsbacken 21, 114 17 Stockholm, Sweden; 2 Department of Immunology, Genetics and Pathology, Uppsala University, 751 85 Uppsala, Sweden; 3 Department of Medicinal Chemistry, Uppsala University, 751 23 Uppsala, Sweden; 4 Science for Life Laboratory, Uppsala University, 751 23 Uppsala, Sweden

**Keywords:** affibody, drug conjugates, hepatic uptake, DM1

## Abstract

Affibody molecules are small affinity-engineered scaffold proteins which can be engineered to bind to desired targets. The therapeutic potential of using an affibody molecule targeting HER2, fused to an albumin-binding domain (ABD) and conjugated with the cytotoxic maytansine derivate MC-DM1 (AffiDC), has been validated. Biodistribution studies in mice revealed an elevated hepatic uptake of the AffiDC, but histopathological examination of livers showed no major signs of toxicity. However, previous clinical experience with antibody drug conjugates have revealed a moderate- to high-grade hepatotoxicity in treated patients, which merits efforts to also minimize hepatic uptake of the AffiDCs. In this study, the aim was to reduce the hepatic uptake of AffiDCs and optimize their in vivo targeting properties. We have investigated if incorporation of hydrophilic glutamate-based spacers adjacent to MC-DM1 in the AffiDC, (Z_HER2:2891_)_2_–ABD–MC-DM1, would counteract the hydrophobic nature of MC-DM1 and, hence, reduce hepatic uptake. Two new AffiDCs including either a triglutamate–spacer–, (Z_HER2:2891_)_2_–ABD–E_3_–MC-DM1, or a hexaglutamate–spacer–, (Z_HER2:2891_)_2_–ABD–E_6_–MC-DM1 next to the site of MC-DM1 conjugation were designed. We radiolabeled the hydrophilized AffiDCs and compared them, both in vitro and in vivo, with the previously investigated (Z_HER2:2891_)_2_–ABD–MC-DM1 drug conjugate containing no glutamate spacer. All three AffiDCs demonstrated specific binding to HER2 and comparable in vitro cytotoxicity. A comparative biodistribution study of the three radiolabeled AffiDCs showed that the addition of glutamates reduced drug accumulation in the liver while preserving the tumor uptake. These results confirmed the relation between DM1 hydrophobicity and liver accumulation. We believe that the drug development approach described here may also be useful for other affinity protein-based drug conjugates to further improve their in vivo properties and facilitate their clinical translatability.

## 1. Introduction

Drug conjugates (DCs) are an emerging class of potent biopharmaceuticals developed to overcome resistance to conventional targeted therapy and reduce off-target toxicity [1,2,3]. DCs are composed of a targeting agent, specifically interacting with a particular antigen, attached to a biologically active drug or cytotoxic compound via a linker. Antibody drug conjugates (ADCs) constitute the most studied class of DCs [3]. Two common types of drug molecules utilized in many ADCs are the auristatins/maytansines that inhibit microtubule polymerization and the calicheamicins which target the minor groove of DNA to induce double-stranded cuts, leading to cell death in both cases. Today, five ADCs have received market approval by the US Food and Drug Administration (FDA); gemtuzumab ozogamicin (Mylotarg^®^), brentuximab vedotin (Adcetris^®^), ado-trastuzumab emtansine (Kadcyla^®^), inotuzumab ozogamicin (Besponsa^®^), polatuzumab vedotin-piiq (Polivy^®^), and many others are still under development or in clinical trials [4,5]. 

Despite the current success, ADCs still face many limitations [6]. Many conjugation strategies rely on unspecific drug attachment to abundant lysine or cysteine residues in the monoclonal antibodies (MAbs). Even though many strategies for site-specific attachment have been developed [7], many ADCs still have a variable drug-to-antibody ratio (DAR) and variable sites of drug attachment, thus forming a nonhomogeneous final product [3,8]. The lack of homogeneity may lead to suboptimal stability, pharmacokinetics, and activity [9]. A random distribution of payloads may potentially interfere with critical residues on the antigen binding regions of MAbs. Moreover, the rather large ADCs may suffer from limited localization and penetration into solid tumors, thus restricting their antitumor efficacy. 

In recent years, alternatives to MAbs have started to emerge. Engineered scaffold proteins (ESPs) are considered the next-generation non-immunoglobulin-based therapeutics [10]. They are derived from small, robust non-immunoglobulin proteins, which are used as “scaffolds” for supporting a surface with the ability to specifically interact with the desired target antigens with high affinity, such as receptors overexpressed on cancer cells. Affibody molecules (6–7 kDa) are one of the most studied classes of ESPs and they are more than 20-fold smaller than MAbs [11,12]. Affibody molecules are based on a 58 aa cysteine-free three-helix scaffold which is derived from one of the IgG binding domains in protein A expressed by Staphylococcus aureus. Affibody molecules have commonly been created by randomization of 13 surface-localized amino acids on helices 1 and 2, followed by phage display selection of binders to different biological targets. Currently, affibody molecules binding with high affinity to several cancer-associated molecular targets, such as human epidermal growth factor receptor 2 (HER2), epidermal growth factor receptor (EGFR), human epidermal growth factor receptor 3 (HER3), insulin-like growth factor 1 receptor (IGF-1R), platelet-derived growth factor receptor beta (PDGFRβ), and carbonic anhydrase 9 (CAIX), have been developed. The cysteine-free structure of affibody molecules permits site-specific conjugation of payloads by introduction of one or more cysteine amino acids at desired position(s) in the scaffold onto which the drug (or any other prosthetic/functional group) can be site-specifically attached. This results in generation of well-defined and homogenous products. The use of affibody molecules as an alternative to MAbs for targeted drug delivery offers several advantages, including efficient production in simple prokaryotic hosts such as *Escherichia coli* [13], efficient and specific drug attachment [14] as well as a relatively smaller size compared to MAbs, which may lead to more efficient penetration and better distribution in solid tumors [15]. However, an important issue for payload delivery using small proteins like affibody molecules is rapid renal excretion. Short in vivo half-life may decrease potency and worsen patient compliance by requiring more frequent administrations. An albumin-binding domain (ABD) was used to prolong the in vivo residence time of affibody molecules by noncovalent interaction with serum albumin [16,17]. We have recently reported on the feasibility of using an anti-HER2 affibody drug conjugate for treatment of HER2-overexpressing tumors in a preclinical murine model [14]. In that study, a HER2-specific affibody molecule, Z_HER2:2891_, was site-specifically conjugated to the antimitotic maytansine derivate (MC-DM1) using maleimide–thiol chemistry. Mice bearing HER2-expressing ovarian cancer xenografts SKOV-3, treated with the tripartite AffiDC, (Z_HER2:2891_)_2_-ABD-MC-DM1, showed significantly longer survival—twice as long compared to mice in control groups. (Z_HER2:2891_)_2_–ABD–MC-DM1 was well-tolerated, and no signs of tissue injury or morphological changes were observed after six cycles of treatment [14]. An interesting finding of that study was the relatively high hepatic uptake of the AffiDC compared to the parental non-MC-DM1-containing HER2-targeting affibody construct. Although no histopathological changes were observed in liver sections of the treated mice, earlier reports indicate that hepatotoxicity may be a serious adverse event associated with several FDA-approved ADCs. For example, it has been observed in several clinical studies involving ado-trastuzumab emtansine (T-DM1) that treatment was associated with elevation of hepatic transaminases and hepatic toxicity [18,19,20]. The mechanism underlying this observed hepatotoxicity remains elusive [20]. A recent report by Yan et al. tried to link hepatic expression of the HER2 receptor to the observed T-DM1-induced hepatotoxicity in a murine model [21]. This study demonstrated that HER2-mediated uptake of T-DM1 by hepatocytes followed by release of DM1 in the cytosol induced several changes, including disorganization of microtubules, nuclear fragmentation, and cell growth inhibition. Even though no liver toxicity was observed in the AffiDC study [14], it is possible that prolonged treatment regimens using higher doses could constitute a problem, and minimization of liver uptake is thus desirable. 

In the initial AffiDC study [14], an attempt to decrease liver uptake was performed by pretreating mice with a several-fold excess of the non-MC-DM1-conjugated, HER2-targeting affibody molecule, Z_HER2:342_, to block available HER2 receptors. However, the hepatic uptake of AffiDC was not reduced by this pretreatment strategy. As mentioned above, the uptake of the AffiDC in liver was significantly higher compared to previously reported HER2-targeting affibody constructs lacking MC-DM1 [16,17]. A possible explanation is that the elevated hepatic uptake is mediated, at least in part, by the presence of the relatively lipophilic MC-DM1. It is known that hydrophobic compounds may facilitate greater reticuloendothelial system clearance and, therefore, increased uptake by the liver. Such effect of drug hydrophobicity on tissue distribution was observed earlier for ADCs, especially at high DARs [22]. 

**Figure 1:**
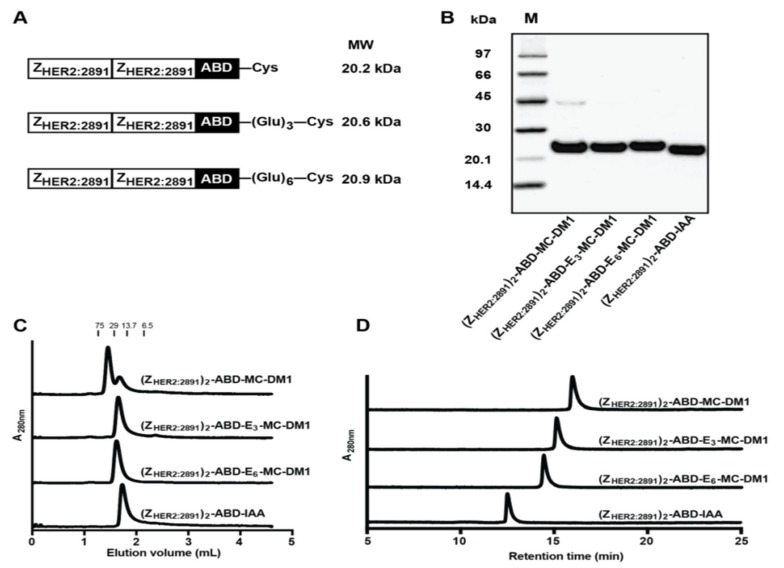
Production and initial biochemical characterization of the conjugates. (**A**) Schematic representation of the proteins. (**B**) Conjugates after final RP-HPLC purification were analyzed on a 4%–12% SDS-PAGE gel under reducing conditions. The numbers to the left are the molecular weight (kDa) of the marker proteins in lane M. (**C**) Analytical size-exclusion chromatography profiles of the conjugates. The numbers above the chromatograms are the molecular weight (kDa) of protein standards. (**D**) RP-HPLC analysis of the conjugates during a 20 min linear gradient from 30% to 60% acetonitrile in water with 0.1% TFA.

In this study, we hypothesized that incorporation of a hydrophilic glutamate-based spacer adjacent to MC-DM1 would reduce hepatic uptake by counteracting the hydrophobic nature of the drug. To test this hypothesis, we designed AffiDCs containing either a triglutamate spacer–((Z_HER2:2891_)_2_–ABD–E_3_–MC-DM1) or a hexaglutamate–spacer–((Z_HER2:2891_)_2_–ABD–E_6_–MC-DM1) (Figure 1A). 

These two drug conjugates were compared, in vitro, with the previously evaluated AffiDC, (Z_HER2:2891_)_2_–ABD–MC-DM1, containing no spacer. The conjugates were also radiolabeled with ^99m^Tc (T_1/2_ = 6 h, Eγ = 140 keV), through the N-terminally localized HEHEHE-tag (Scheme 1 in Supplementary Figure S1), and the influence of the glutamate spacer on hepatic uptake and overall biodistribution in a HER2-overexpressing preclinical murine tumor model was investigated.

## 2. Results

### 2.1. Production and Biochemical Characterization of the Affibody–MC-DM1 Conjugates

The affibody constructs, schematically represented in Figure 1A, were recombinantly expressed and purified, and MC-DM1 was conjugated to a C-terminal cysteine. A construct lacking MC-DM1 was used ((Z_HER2:2891_)_2_–ABD–IAA) as a control, where the C-terminal cysteine was instead alkylated by 2-iodoacetamide (IAA). The purified conjugates were analyzed by SDS-PAGE under reducing conditions, and the gel showed pure proteins with essentially the expected molecular weights (Figure 1B). A weak contaminating band in the lane of (Z_HER2:2891_)_2_–ABD–MC-DM1 was visible with a molecular weight of approximately 45 kDa, and could thus constitute a dimer. The conjugates were further analyzed by size-exclusion chromatography under native conditions. The chromatogram from (Z_HER2:2891_)_2_–ABD–MC-DM1 showed that the protein was eluted as a double-peak, where the major peak had a retention time corresponding to a dimer and the minor peak had a retention time corresponding to a monomer. The other three conjugates were eluted as a single symmetrical peak with a retention time corresponding to a monomer (Figure 1C). The molecular weights were measured by ESI-TOF (Table 1) and the results showed conjugates matching exactly the molecular weight of monomeric proteins with a drug-to-affibody ratio of 1. The conjugates were further analyzed by passage through a C18 column using a linear gradient of acetonitrile in water in an RP-HPLC setup (Figure 1D). The recorded chromatograms showed that (Z_HER2:2891_)_2_–ABD–E_6_–MC-DM1 was eluted first, followed by (Z_HER2:2891_)_2_–ABD–E_3_–MC-DM1 and (Z_HER2:2891_)_2_–ABD–MC-DM1, suggesting that incorporation of glutamate residues reduced the hydrophobicity of the conjugates by shielding the MC-DM1 part from interaction with the C18 column. The control (Z_HER2:2891_)_2_–ABD–IAA, lacking MC-DM1, was eluted even earlier than the other three, further suggesting a profound increase in hydrophobicity of the conjugates by addition of MC-DM1.

### 2.2. Binding Specificity and Affinity Determination of Affibody–MC-DM1 Conjugates 

**Figure 2:**
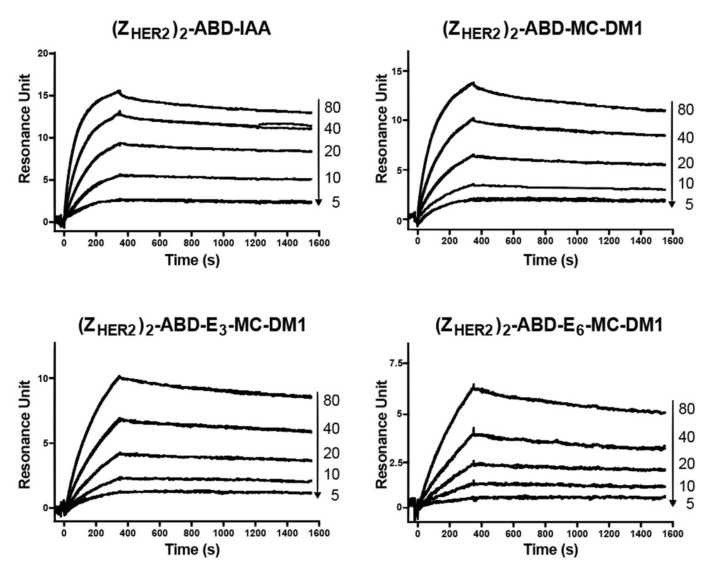
Biosensor analysis of the interactions between the conjugates and HER2. Dilution series of the conjugates were sequentially injected over flow cells with immobilized extracellular domain of HER2. All experiments were repeated once and each panel is an overlay of all concentrations, in duplicates, for each conjugate. The numbers to the right of each panel indicate the concentrations of the injected conjugates (nM) corresponding to each sensorgram.

**Figure 3:**
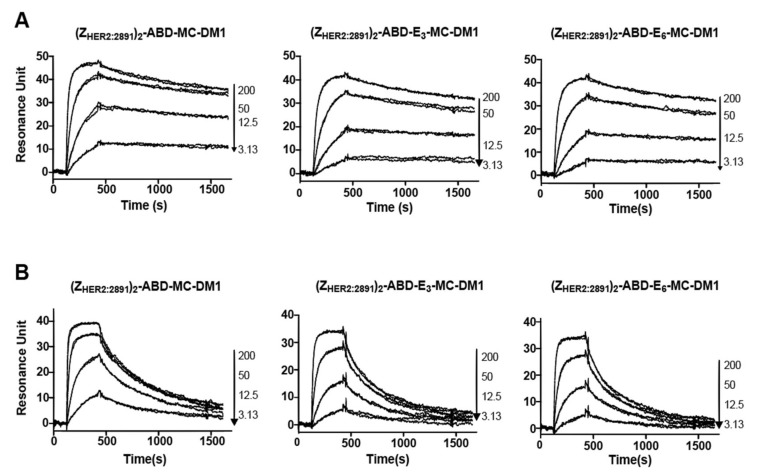
Biosensor analysis of the interactions between the conjugates and serum albumin. Serial dilutions of the conjugates were injected over a flow cell with immobilized HSA (**A**) or mouse serum albumin (MSA) (**B**). All experiments were repeated once, and each panel is an overlay of all concentrations in duplicates for each conjugate. The numbers to the right of each panel indicate the concentrations of the injected conjugates (nM) corresponding to each sensorgram.

To investigate if MC-DM1 conjugation and glutamic acid insertion would affect the affinity of Z_HER2:2891_ to HER2, a dilution series of the conjugates were injected into a biosensor over three different surfaces with different levels of immobilized extracellular domain of HER2 (Figure 2). Since each construct contains two affibody molecules, a potential avidity effect could occur if the HER2 receptor molecules are too closely spaced and allow simultaneous interaction with both. The interaction was analyzed assuming a 1:1 interaction, and consistent on- and off-rates were determined from the recorded sensorgrams for the three surfaces, indicating a lack of avidity effect and that a 1:1 interaction occurred. The equilibrium dissociation constant (K_D_) for the interactions were determined from the on- and off-rates and are displayed in Table 2. The K_D_ values were found to be similar for the three MC-DM1 conjugates and the control, and ranged from 17 to 28 nM. The ability of the conjugates to interact with human serum albumin (HSA) and mouse serum albumin (MSA) was investigated by injection of a dilution series over a chip with immobilized HSA or MSA (Figure 3). The kinetic constants were derived from the sensorgrams (Table 3). The affinities (K_D_) for HSA ranged from 0.57 to 1.2 nM. The affinities for MSA were slightly weaker and ranged from 2.5 to 8.0 nM.

### 2.3. In Vitro Cytotoxicity Analysis 

**Figure 4:**
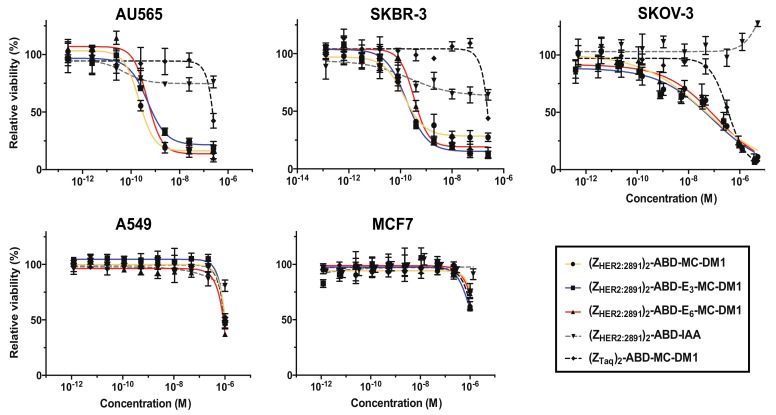
In vitro cytotoxicity of the conjugates. The cytotoxicity was determined by incubating serial dilutions of the conjugates with the cell lines indicated above the panels. The concentration ranges were 0.25–250 nM (AU565), 0.13–250 nM (SKBR3), 0.4 nM–5 μM (SKOV3), 1.2 nM–1 μM (A549), and 1.2 nM–1.35 μM (MCF7). The relative viability of the cells is plotted on the *Y*-axis as a function of the compound concentration on the *X*-axis. The relative viability of cells cultivated in medium was used as reference (100%). Each datapoint corresponds to the average of four independent experiments and the error bars correspond to 1 SD.

The cytotoxicity of the affibody–MC-DM1 conjugates was measured by treating AU565 (high HER2 expression), SKBR3 (high HER2 expression), SKOV3 (high HER2 expression), A549 (moderate HER2 expression), and MCF7 (low HER2 expression) cells, with serial dilutions of the conjugates followed by measurement of cell viability (Figure 4, Table 4). Two controls were also included, the nontoxic control (Z_HER2:2891_)_2_–ABD–IAA lacking MC-DM1, and the nontarget control (Z_Taq_)_2_–ABD–MC-DM1. The nontarget control was a size matched control where Z_HER2:2891_ had been replaced with Z_Taq_, an affibody molecule that specifically binds to DNA polymerase from *Thermus aquaticus*, and was thus not expected to bind to any protein of human origin [14]. (Z_Taq_)_2_–ABD–MC-DM1 was previously characterized and was found to be a homogenous protein of the expected molecular weight with a purity >95% [14]. It was found not to interact with the HER2 receptor and did not induce cell death in cells overexpressing the HER2 receptor [14]. The targeting drug conjugates demonstrated subnanomolar IC_50_ values on AU565 and SKBR-3 cell lines. For AU565 cells, the IC_50_ values ranged from 0.22 to 0.48 nM, and for SKBR3 cells from 0.14 to 0.38 nM. For SKOV3, the IC_50_ values ranged from 47 to 116 nM. The nontoxic control (Z_HER2:2891_)_2_–ABD–IAA showed a slight inhibition of cell growth on the AU565 and SKBR3 cell lines at higher concentrations (>10^−9^ M). For SKOV3 cells, a slight growth-promoting effect was observed at the highest concentration. All conjugates demonstrated a substantially weaker cytotoxic effect on A549 and MCF7 cells. The IC_50_ could not be measured at the concentrations used, but from Figure 4, it is evident that they were weaker than 10^−6^ M in all cases. For all five cell lines, the nontarget control (Z_Taq_)_2_–ABD–MC-DM1 required high concentrations to affect cell viability. The IC_50_ values could not be determined from the concentration range used, except for SKOV3 cells (IC_50_ 350 nM). From Figure 4, it is evident that the IC_50_ value is 2 to 3 orders of magnitude weaker for the high expressing cell lines. The nontarget control (Z_Taq_)_2_-ABD-MC-DM1 had a cytotoxic potential similar to the Z_HER2:2891_-containing conjugates on A549 and MCF7 cells. 

### 2.4. Radiolabeling and Stability Test of Radiolabeled Constructs

For further in vitro characterization and to facilitate in vivo comparison, the conjugates were site-specifically radiolabeled with ^99m^Tc through the N-terminally localized HEHEHE-tag. Data concerning the labeling yield, radiochemical purity, and stability of the conjugates are presented in Table 5. All three AffiDCs were efficiently labeled with ^99m^Tc (radiochemical yield = 58%–61%). The radiochemical purity after purification by size-exclusion chromatography was >99%. Incubation with a 5000-fold molar excess of histidine showed that most of the activity (>97%) was still bound to the AffiDCs even after 24 h (Table 5).

### 2.5. In Vitro Specificity and Internalization 

**Figure 5:**
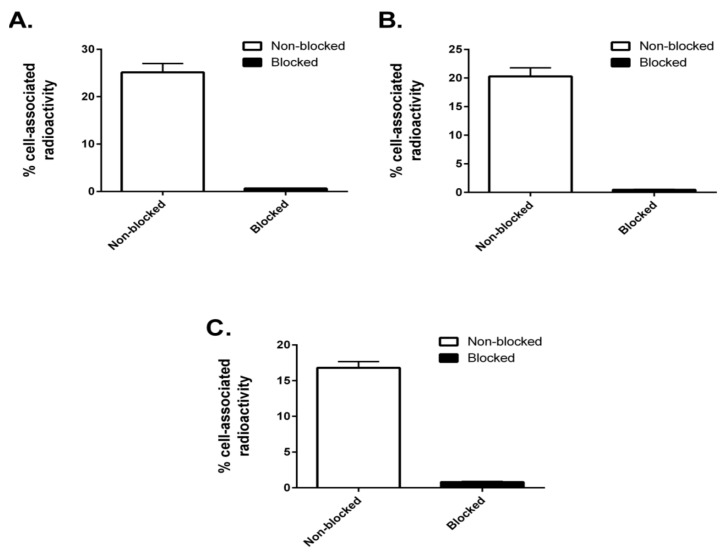
In vitro specificity. Specificity of binding of ^99m^Tc-labeled (Z_HER2:2891_)_2_–ABD–MC-DM1 (**A**), (Z_HER2:2891_)_2_–ABD–E_3_–MC-DM1 (**B**), and (Z_HER2:2891_)_2_–ABD–E_6_–MC-DM1 (**C**) to HER2-expressing SKOV-3 cells in vitro. Each bar shows the mean of the values measured in 3 dishes and the error bars correspond to SD.

To evaluate the integrity and cell interaction capability of the radiolabeled constructs, a specificity test was conducted. SKOV3 cells were incubated with the conjugates, with or without preincubation with a 500-fold molar excess of nonradiolabeled anti-HER2 affibody molecule Z_HER2:342_ to block available HER2 receptors. Z_HER2:342_ binds to the same epitope as Z_HER2:2891_ [23]. The three constructs could bind to SKOV3 cells in a HER2-dependent manner, since the cell-associated radioactivity was reduced significantly when HER2-receptors were presaturated with Z_HER2:342_ (Figure 5).

**Figure 6:**
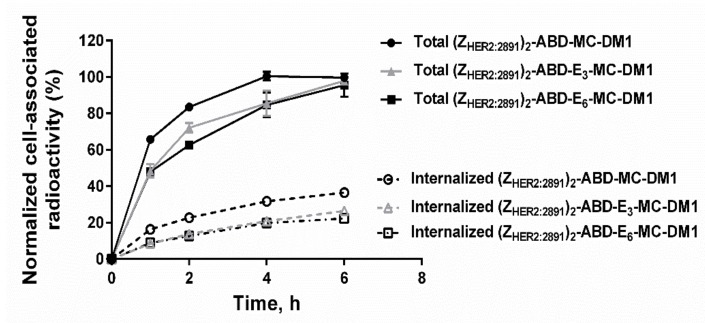
In vitro internalization. Internalization of ^99m^Tc-labeled (Z_HER2:2891_)_2_–ABD–MC-DM1 (circle), (Z_HER2:2891_)_2_–ABD–E_3_–MC-DM1 (triangle), and (Z_HER2:2891_)_2_–ABD–E_6_–MC-DM1 (square) by HER2-expressing SKOV-3 cells at 37 °C. Each datapoint is the average of three individual experiments ± 1 SD.

The internalization of the three AffiDCs by SKOV3 cells (high HER2 expression) was performed using a continuous incubation assay (Figure 6). The cell-associated radioactivity showed a continuous growth for the three AffiDCs up to 6 h of incubation, but at slightly different rates. The internalization of the three AffiDCs also increased over time but, again, at different rates. The construct with no glutamate spacer (Z_HER2:2891_)_2_–ABD–MC-DM1 demonstrated the highest rate of internalization compared to the glutamate spacer-containing variants (Z_HER2:2891_)_2_–ABD–E_3_–MC-DM1 and (Z_HER2:2891_)_2_–ABD–E_6_–MC-DM1 at all studied timepoints. The internalized fraction after 6 h incubation accounted for 36.5% ± 1.2%, 26.4% ± 1.3%, and 22.3% ± 2.3% of the total cell-associated radioactivity for (Z_HER2:2891_)_2_–ABD–MC-DM1, (Z_HER2:2891_)_2_–ABD–E_3_–MC-DM1, and (Z_HER2:2891_)_2_–ABD–E_6_–MC-DM1, respectively (Figure 6).

### 2.6. Biodistribution and In Vivo Tumor Targeting

**Figure 7:**
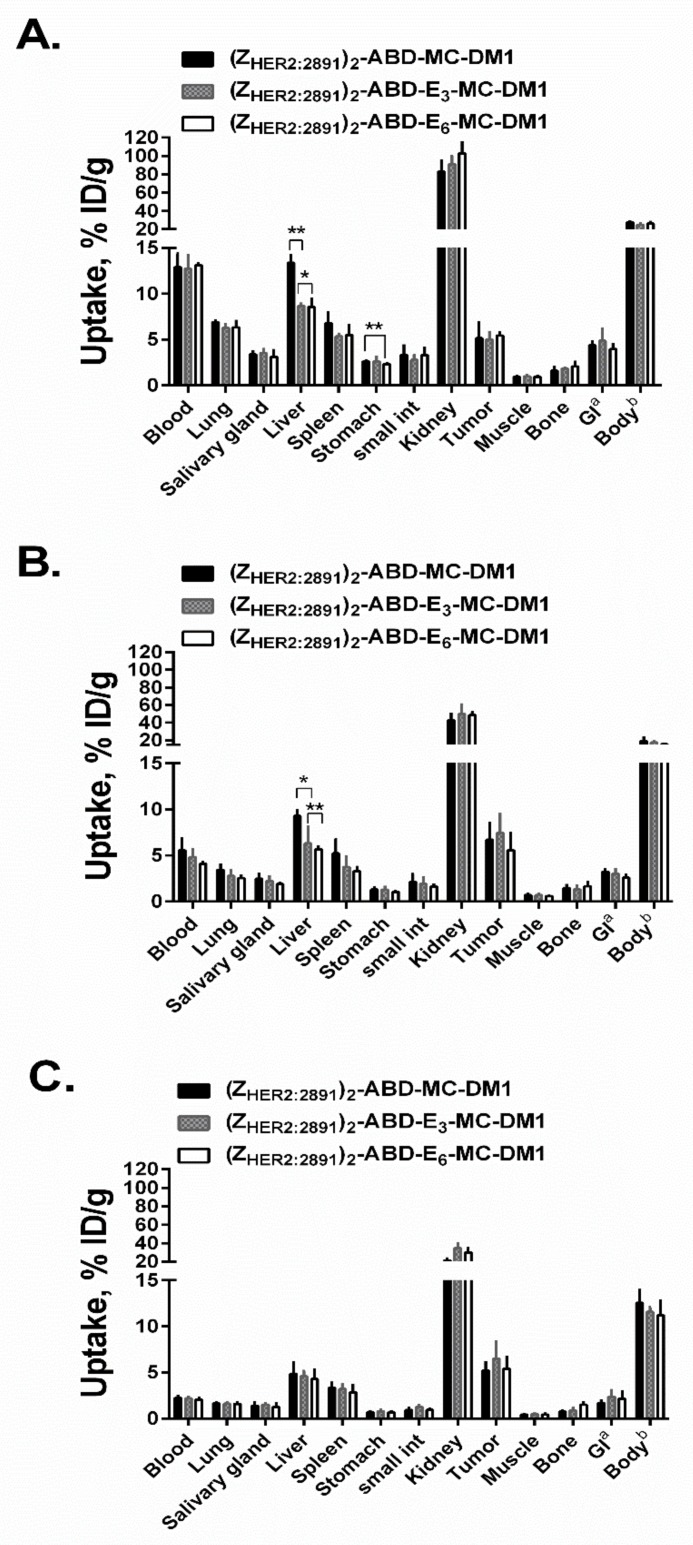
In vivo biodistribution. Comparative biodistribution of ^99m^Tc-labeled DM1 conjugates expressed as % ID/g and presented as an average value from 4 animals ± 1 SD at 4 (**A**), 24 (**B**), and 46 (**C**) h post i.v. injection in female BALB/c nude mice bearing SKOV-3 xenografts. a,b Data are presented as % ID per whole sample. Data were assessed by one-way ANOVA with Bonferroni’s post hoc multiple comparisons test in order to determine significant differences between groups (*p* < 0.05) at the same timepoint.

Data concerning in vivo biodistribution and tumor targeting of ^99m^Tc-labeled (Z_HER2:2891_)_2_–ABD–MC-DM1, (Z_HER2:2891_)_2_–ABD–E_3_–MC-DM1, and (Z_HER2:2891_)_2_–ABD–E_6_–MC-DM1 at 4, 24, and 46 h post injection (p.i.) in BALB/c-nu/nu mice bearing HER2-expressing SKOV-3 xenografts are displayed in Figure 7. There was no significant difference in the residence in circulation between the AffiDCs at all studied timepoints. The tumor uptake of the three AffiDCs was comparable at all studied timepoints and showed better retention with time compared to uptake in other organs. By 46 h p.i., the tumor uptake of all three AffiDCs (5.2%–6.5% ID/g) was higher than the uptake in any other organ except the kidneys. The tumor uptake at 46 h p.i. in mice bearing RAMOS lymphoma xenografts (HER2 negative) was 6–10-fold lower compared to that in SKOV-3 xenografts: 0.9% ± 0.1%, 0.6% ± 0.1%, and 0.6% ± 0.2% ID/g for (Z_HER2:2891_)_2_–ABD–MC-DM1, (Z_HER2:2891_)_2_–ABD–E_3_–MC-DM1, and (Z_HER2:2891_)_2_–ABD–E_6_–MC-DM1, respectively (Figure S2).

There was no significant difference in activity concentration in most organs, and it generally followed the kinetics in the blood. However, a striking difference in the uptake in the liver was observed. The activity uptake of (Z_HER2:2891_)_2_–ABD–E_3_–MC-DM1 and (Z_HER2:2891_)_2_–ABD–E_6_–MC-DM1 was significantly lower compared to (Z_HER2:2891_)_2_–ABD–MC-DM1 at 4 h p.i. (8.7% ± 0.2% and 8.6% ± 0.9% vs. 13.4% ± 0.9 % ID/g) and at 24 h p.i. (6.3% ± 1.8% and 5.7% ± 0.3% vs. 9.3% ± 0.7% ID/g). This difference in hepatic uptake disappeared by 46 h p.i. (6.5% ± 1.8% and 5.4% ± 1.3% vs. 5.2% ± 0.9% ID/g). Interestingly, there was no significant difference in radioactivity uptake in the gastrointestinal tract and kidneys, in connection with the reduction in hepatic uptake. 

## 3. Discussion

In this study, the aim has been to investigate if hepatic uptake of AffiDCs could be reduced by incorporation of a hydrophilic glutamate-based spacer adjacent to site of MC-DM1 attachment. Hepatotoxicity is one of the most common reasons for drug development failures and withdrawal of drugs from the market [24,25]. In the field of ADCs, several reports have found a link between treatment and drug-induced liver injuries. For example, it was observed that T-DM1 therapy was associated with serious grade 3 or greater adverse events in some patients, including hepatotoxicity [18,19,20]. Similarly, several patients treated with the prostate-specific membrane antigen-directed ADC, MLN2704, have experienced elevated dose-dependent levels of hepatic transaminases [26]. Many drug development programs therefore include development of methods aiming to identify potential liver toxicities and their mechanisms [20,21,24,25]. Despite those efforts, hepatotoxicity still remains to be one of the most complex and poorly understood areas of human toxicity. For example, Yan and coworkers tried to understand the molecular basis for hepatotoxicity induced by T-DM1 [21]. This group concluded that HER2-mediated uptake of T-DM1 by hepatocytes is directly linked to DM1-associated liver toxicity.

We have earlier reported on the development of an AffiDC, (Z_HER2:2891_)_2_–ABD–MC-DM1, targeting HER2. The AffiDC demonstrated relatively high hepatic uptake in mice post i.v. injection. The accumulation in liver of AffiDC was several-fold higher compared to other ABD-fused affibody molecules [16,17]. As mentioned above, Yan et al. reported earlier that T-DM1 induced liver toxicity through a HER2-mediated uptake of the ADC by hepatocytes. We tested this assumption by preinjecting mice with >100-fold molar excess of parental HER2-targeting affibody molecule to potentially block available HER2 receptors [14]. We found that there was no reduction in hepatic uptake of AffiDC after HER2-blocking, suggesting an unspecific liver uptake of AffiDC [14]. The main difference between AffiDC and other reported ABD-fused affibody molecules [16,17] is the presence of the drug DM1. Such drug-induced hepatic uptake has also been observed for MAbs after addition of the drug molecules [27,28]. Several groups have hypothesized that the increased hepatic uptake of ADCs may result from an increase in overall hydrophobicity of the conjugate after addition of lipophilic linkers or drug molecules [22,27,28]. Based on this, it would therefore be reasonable to suspect that the relatively high hepatic uptake of AffiDC is mainly a drug-mediated effect. We hypothesized that the incorporation of a hydrophilic spacer consisting of glutamic acid residues next to the cysteine used for MC-DM1 conjugation would lead to a decrease in hepatic uptake.

Comparison of (Z_HER2:2891_)_2_–ABD–MC-DM1 and the nondrug-conjugated (Z_HER2:2891_)_2_–ABD–IAA showed that addition of MC-DM1 increased the retention time during passage through a RP-HPLC column (Figure 1C). This represents evidence of the increased hydrophobicity conferred by MC-DM1. Further comparison of (Z_HER2:2891_)_2_–ABD–MC-DM1 with the newly designed polyglutamate spacer-containing variants, (Z_HER2:2891_)_2_–ABD–E_3_–MC-DM1 and (Z_HER2:2891_)_2_–ABD–E_6_–MC-DM1, showed that addition of glutamic acid residues decreased the retention time, suggesting a shielding effect on the interaction with the C18 ligand in the column. 

The newly designed AffiDCs demonstrated high binding affinity as well as specificity to HER2 receptors (Figure 2). Retaining the capacity to bind HER2 with high affinity is essential for efficient targeting. The setup in the biosensor with immobilized receptor only allows for determination of an apparent affinity since the affinity of the two affibody domains in the AffiDC for HER2 could be different, and we would thus record a mixture of the signal obtained from affibody one and affibody two interacting with HER2. However, since the kinetic constants were similar for the AffiDC/HER2 interaction on three surfaces with different HER2 density, only one of them are engaged with HER2 at any given time, and an avidity in the interaction is between the analyte and the surface is negligible. The setup with immobilized HER2 rather than immobilized AffiDC was chosen since it better mimics the cell experiments where HER2 is part of the plasma membrane and the AffiDC is free in solution. The albumin-binding function was also retained as demonstrated by the biosensor analysis of the interactions between the conjugates and serum albumin (Figure 3). All tested conjugates demonstrated a sub- to single-digit nanomolar affinity (K_D_ value) for both HSA and MSA. These K_D_ values are similar to results obtained previously for several affibody-based ABD-fused targeting agents [14,16,17,29,30]. The biodistribution experiments confirmed the capacity of ABD to extend AffiDC circulation time. The three AffiDCs demonstrated comparable retention in the blood at all studied timepoints. The blood associated radioactivity was 13% ± 1%, 5% ± 1%, and 2% ± 0.2% ID/g at 4, 24, and 46 h p.i. of the ^99m^Tc-labeled AffiDCs. Affibody molecules, by themselves or as head-to-tail dimers, are generally cleared almost completely from blood within 1 h [31]. For example, in a similarly conducted biodistribution experiment, the blood activity 4 h p.i. of an anti-HER2 monomeric Z and dimeric ZZ affibody molecules (lacking an ABD) was only 1.5% ± 0.2% and 2.5% ± 0.2 % ID/g, respectively.

Being a natural amino acid, inserted glutamates were not expected to affect the degradation of affibody–MC-DM1 conjugates in the lysosomes during the process of cell intoxication. The results from the in vitro toxicity study demonstrated clearly the cytotoxicity potential of the newly designed AffiDCs with IC_50_ values similar to the parental (Z_HER2:2891_)_2_–ABD–MC-DM1 (Figure 4 and Table 4). This cell killing potential is also comparable to that of the clinically approved trastuzumab emtansine, as was demonstrated earlier [14]. It is evident that HER2 specificity is important for efficient cytotoxic activity of the AffiDCs. The sensitivity of the low-HER2-expressing MCF-7 cells and the moderate-HER2-expressing A549 cells for AffiDCs was almost 3 orders of magnitude lower than the sensitivity of the high-HER2-expressing SKOV3, SKBR3, and AU565 cell lines. Surprisingly, there was a big discrepancy between the sensitivity of the high-HER2-expressing cell lines to our AffiDCs. The measured IC_50_ values were in the range of 47 to 116 nM in SKOV-3 cells while it was ca. 300-fold lower in SKBR3 and AU565 (Table 4). As the level of HER2 in the three cell lines is comparable, the difference may be attributed to other factors known to decrease sensitivity to drug conjugates. These may include, among others, differences in the expression level of multidrug resistance transporters, impairment of receptor internalization, and dysfunction of lysosomal degradation mechanisms [32,33,34]. 

An unexpected finding of this study was the growth-promoting effect for SKOV-3 cells observed after incubation with the non-DM1-containing (Z_HER2:2891_)_2_–ABD–IAA affibody. We may speculate that it might be caused by HER2 dimerization, mediated by the two affibody domains in the construct, followed by an increase in intracellular signaling by the receptor. It is possible that the increased proliferation observed during incubation with a high concentration of (Z_HER2:2891_)_2_–ABD–IAA could enhance the cytotoxic activity of DM1, since the drug is strongly acting/selective towards rapidly dividing cells through prevention of microtubule formation.

The three AffiDCs were site-specifically labeled with ^99m^Tc through the N-terminally localized HEHEHE-tag (Table 5). After histidine challenge for 24 h, most (>97%) of the radioactivity was still associated with the conjugates. Stable labeling of the conjugates is a perquisite for accurate in vivo evaluation. It is important to mention that the spacer in (Z_HER2:2891_)_2_–ABD–E_3_–MC-DM1 and (Z_HER2:2891_)_2_–ABD–E_6_–MC-DM1 could potentially offer an alternative weak-chelating pocket for ^99m^Tc, due to the electron-donating properties of glutamate sidechains [35]. However, the minimal activity release in the presence of competing histidines revealed that this is not the case for these conjugates.

The three radiolabeled AffiDCs demonstrated HER2-mediated binding to SKOV-3 cells in vitro (Figure 5). This clearly showed that site-specific radiolabeling had no negative influence on the HER2-binding properties. There was an apparent influence of the spacer on the internalization rate of the conjugates where both conjugates containing a polyglutamate spacer demonstrated a slower internalization rate compared to (Z_HER2:2891_)_2_–ABD–MC-DM1 at all studied timepoints (Figure 6). Nonetheless, the internalization experiment clearly showed that both (Z_HER2:2891_)_2_–ABD–E_3_–MC-DM1 and (Z_HER2:2891_)_2_–ABD–E_6_–MC-DM1 are still efficiently internalized and should thus be capable of targeted delivery of the drug DM1 to kill tumor cells similar to the previously evaluated (Z_HER2:2891_)_2_–ABD–MC-DM1.

The biodistribution data of the three AffiDCs in BALB/c nu/nu mice were in a good agreement with the data reported earlier for (Z_HER2:2891_)_2_–ABD–MC-DM1 [14]. The AffiDCs clearly demonstrated the capacity to bind to tumor xenografts in vivo in a HER2-dependent manner (Figure 7 and Figure S2). The results of the biodistribution experiment confirmed the relation between the hydrophobicity of the DM1-containing AffiDC and liver accumulation. Incorporation of the hydrophilic polyglutamate spacer enabled modulation of liver accumulation. The hydrophilized (Z_HER2:2891_)_2_–ABD–E_3_–MC-DM1 and (Z_HER2:2891_)_2_–ABD–E_6_–MC-DM1 AffiDCs had nearly 1.5-fold (*p* < 0.05) lower liver accumulation than that of the parental (Z_HER2:2891_)_2_–ABD–MC-DM1 (Figure 7). Several overlapping factors may be associated with the selective accumulation of drug conjugates in the liver [36]. These factors include affinity between the construct and the hepatocellular transport proteins residing outside of the cells, the potential to trigger endocytosis, the release from the endosomes or lysosomes inside the hepatic cells, and the rate at which the linker between the targeting agent and the drug is cleaved. Moreover, the affinity between the construct and its catabolites to the hepatocellular efflux transporters might also play a role in hepatic accumulation. It is important to mention that the radiolabel and the drug DM1 are located at different ends of the AffiDCs. This makes it difficult to link any observed differences in hepatic accumulation to the nature of DM1–catabolites formed after lysosomal degradation. The most plausible explanation for the observed difference in hepatic accumulation of radioactivity, stems from the difference in uptake of the three AffiDCs—having different degree of hydrophilicity—by hepatocytes. This is based on earlier findings, where reduction of overall hydrophobicity of targeting agents was found to suppress hepatic uptake [29,37,38,39]. Decreasing overall hydrophobicity by incorporation of hydrophilic groups or linkers has also resulted in better in vivo targeting properties for bulky ADCs, particularly reduction of hepatic accumulation [22,28]. Since the AffiDCs are approximately 10 times smaller than ADCs, it is expected that the influence of hydrophilization on liver uptake should be more profound for AffiDCs. Surprisingly, the effect on hepatic accumulation was not directly proportional to the number of incorporated glutamate residues and no significant difference in liver accumulation between (Z_HER2:2891_)_2_–ABD–E_3_–MC-DM1 and (Z_HER2:2891_)_2_–ABD–E_6_–MC-DM1 was found at any of the timepoints (Figure 7). Regardless of the underlying reason, a reduction in hepatic uptake could have a positive impact on the maximum tolerated dose of AffiDC. 

## 4. Materials and Methods

All chemicals were purchased from Sigma-Aldrich (St. Louis, MO, USA) or Merck (Darmstadt, Germany) unless otherwise stated. Restriction enzymes were from New England Biolabs (Ipswitch, MA, USA). 

### 4.1. Construction of Genes Encoding Affibody Constructs

Genes encoding (Z_HER2:2891_)_2_–ABD–Cys and (Z_Taq_)_2_–ABD–Cys were constructed previously [14]. Genes encoding (Z_HER2:2891_)_2_–ABD–E_3_–Cys, (Z_HER2:2891_)_2_–ABD–E_6_–Cys flanked by *Nde*I and *Bam*HI restriction sites were synthesized by Thermo Fisher Scientific (Waltham, MA, USA). They were subcloned into the pET-21a(+) plasmid vector (Novagen, Madison, WI, USA) using *Nde*I and *BamH*I restriction enzymes.

### 4.2. Expression and Purification of Affibody Constructs 

The affibody constructs were expressed at 37 °C in shake flask cultures of *Escherichia coli* BL21 Star (DE3) (New England Biolabs). When OD_600_ was between 0.6 and 1, protein expression was induced by addition of 1 mM isopropyl β-D-1-thiogalactopyranoside (Appolo Scientific, Stockport, UK). Protein production was carried out for 3 h, after which the cells were harvested by centrifugation and lysed by sonication. The supernatants were clarified by centrifugation and filtration through a 0.45 μm Acrodisc syringe filter (Pall, Port Washington, NY, USA). The recombinantly expressed affibody constructs were purified by affinity chromatography on a HiTrap NHS sepharose column (GE Healthcare, Uppsala, Sweden) with immobilized human serum albumin (HSA) using an ÄKTA system (GE Healthcare), essentially as previously described [14] including elution with 50 mM acetic acid. The fractions containing affibody constructs were pooled and lyophilized. 

### 4.3. Conjugation with MC-DM1 

The lyophilized proteins were dissolved in PBS at pH 6.5 to a final concentration of 0.1 mM and incubated with 5 mM tris(2-carboxyethyl) phosphine (TCEP) for 30 min at room temperature., to reduce the sulfur on the C-terminal cysteine of the constructs, which could potentially have been oxidized during protein production and purification. Freshly prepared MC-DM1 (Levena Biopharma, San Diego, CA, USA), dissolved in DMSO (20 mM), was mixed with the affibody constructs at a molar ratio of 2:1, and the conjugation mixture was incubated overnight at r.t. The conjugation reaction mixture was diluted with HPLC buffer A (0.1% trifluoroacetic acid in H_2_O) and then loaded on a Zorbax C18 SB column (Agilent, Santa Clara, CA, USA). Bound material was eluted by a 25 min gradient from 20% or 30% to 60% or 80% buffer B (0.1% trifluoroacetic acid in acetonitrile). The fractions containing affibody–MC-DM1 conjugates were pooled followed by lyophilization. 

Capping of the C-terminal cysteine to create the nontoxic control (Z_HER2:2891_)_2_–ABD–IAA was carried out with 2-iodoacetamide. Lyophilized (Z_HER2:2891_)_2_–ABD–Cys was dissolved in alkylation buffer (6M urea, 0.1 M NH_4_HCO_3_) after which dithiothreitol was added to a final concentration of 4 mM, followed by incubation for 30 min at 37 °C to reduce any potentially oxidized cysteine residues. 2-Iodoacetamide was added to a final concentration of 10 mM followed by incubation for 30 min at r.t. to alkylate the cysteines. The capped proteins were purified by RP-HPLC as described above for the affibody–MC-DM1 conjugates, followed by lyophilization.

The lyophilized proteins were dissolved in sterile PBS buffer and stored at −20 °C until use. Purified proteins (5 μg in each sample) were analyzed by SDS-PAGE (Biorad, Hercules, CA, USA) under reducing conditions. The molecular weight of purified affibody–MC-DM1 conjugates was measured by ESI-TOF mass spectrometry (Agilent).

### 4.4. Binding Specificity and Affinity Determination 

A Biacore T200 and a Biacore 3000 instrument (GE Healthcare) were used for biosensor analysis. The extracellular domain of HER2 (HER2_ECD_) (Sino Biological, Beijing, China) was immobilized to 210, 310, and 456 RUs on three different flow cells on a CM5 chip by amine coupling in sodium acetate buffer, pH 4.5. A reference flow cell was created by activation and deactivation. On a second CM-5 chip, HSA (Novozymes, Bagsvaerd, Denmark), MSA (Sigma-Aldrich, St. Louis, MO, USA), and BSA (Merck Millipore) were immobilized in the same way. The final immobilization levels were 869, 584, and 779 RUs, respectively. HBS-EP (10 mM HEPES, 150 mM NaCl, 3 mM EDTA, 0.05% Tween 20, pH 7.4) was used as running buffer and for dilution of the analytes. All experiments were performed at 25 °C with a flow rate of 50 μL/min. The chips were regenerated by injection of 15 mM HCl for 30 s. The binding kinetics was analyzed by the Biacore evaluation software using the one-to-one kinetics model.

### 4.5. In Vitro Cytotoxicity Analysis 

AU565, SKBR-3, SKOV-3, A549, and MCF7 cell lines were obtained from American Type Culture Collection (American Type Culture Collection, ATCC via LGC Promochem, Borås, Sweden) and were grown in McCoy’s 5A (SKOV-3, SKBR-3), RPMI-1640 (AU565), or Dulbecco’s modified Eagle medium (A549 and MCF7) (Flow, Irvine, UK) supplemented with 10% FBS (Sigma-Aldrich, St. Louis, MO, USA) in a humidified incubator at 37 °C in 5% CO_2_ atmosphere. Approximately 5000 cells/well (2000 cells/well for SKOV-3) were seeded in a 96-well plate and allowed to attach for 24 h. Subsequently, the medium was replaced with fresh medium containing serial dilutions of affibody–MC-DM1 conjugates or 2-iodoacetamide-capped nontoxic control followed by incubation for 72 h. Cell viability was determined using Cell Counting Kit-8 (CCK-8; Sigma-Aldrich) according to the manufacturer’s protocol with measurement of A_450_ in each well. The obtained absorbance values were analyzed by GraphPad Prism using a log(inhibitor) vs. response-variable slope (four parameters) model (GraphPad Software, Inc., La Jolla, CA, USA).

### 4.6. Radiolabeling and Stability Test of Radiolabeled Constructs

Site-specific radiolabeling of AffiDCs ((Z_HER2:2891_)_2_–ABD–MC-DM1, (Z_HER2:2891_)_2_–ABD–E_3_–MC-DM1, and (Z_HER2:2891_)_2_–ABD–E_6_–MC-DM1) with ^99m^Tc using (^99m^Tc(CO)_3_(H_2_O)_3_) + precursor was performed as previously described [14]. In brief, eluted pertechnetate, ^99m^TcO_4_^-^, (400–500 μL) from ^99^Mo/^99m^Tc generator was added to a CRS kit (PSI, Villigen, Switzerland) to generate the (^99m^Tc(CO)_3_(H_2_O)_3_) + (tricarbonyl technetium) precursor. The mixture was vortexed carefully and incubated at 100 °C for 20 min. After incubation, 20 μL of the tricarbonyl technetium solution was added to a tube containing 55 μg of the respective AffiDC in 100 μL of PBS and incubated for 60 min at 60 °C. To isolate the radiolabeled AffiDCs, the mixture was passed through a NAP-5 size-exclusion column (GE Healthcare) pre-equilibrated and eluted with 2% BSA in PBS. Radiochemical yield and purity of the conjugates were determined using silica-impregnated ITLC strips (150–771 DARK GREEN Tec-Control Chromatography strips (Biodex Medical Systems, Shirley, NY, USA) eluted with PBS and measured using the Cyclone Storage Phosphor System (PerkinElmer, Waltham, MA, USA). To evaluate the stability of the radiolabeled AffiDCs, they were incubated with a 5000-fold molar excess of histidine at 37 °C for up to 4 and 24 h, respectively. The percentage of protein-bound radioactivity after histidine challenge was determined using radio-ITLC as mentioned above.

### 4.7. In vitro Specificity and Internalization

To confirm the specificity of binding of ^99m^Tc-radiolabeled AffiDCs to HER2-expressing cells in vitro, SKOV-3 cells (5–7.5 × 10^5^) were incubated with 2 nM of each conjugate at 37 °C for 60 min (*n* = 3). For blocking, another set of dishes containing SKOV-3 cells were preincubated with 500-fold molar excess of nonlabeled anti-HER2 affibody molecule Z_HER2:342_ prior to the addition of radiolabeled AffiDCs. Thereafter, both medium and cells were collected from each dish and measured for radioactivity using an automated γ-spectrometer (1480 Wizard; Wallac Oy, Turku, Finland). Data are presented as mean values from three cell dishes with standard deviation.

The internalization of ^99m^Tc-radiolabeled AffiDCs by HER2-expressing cells was studied using a method described earlier by Altai et al. [14]. For this, four groups of dishes (*n* = 3) containing SKOV-3 cells (5–7.5 × 10^5^ cells/dish) were incubated with 2 nM (per dish) of the respective conjugate at 37 °C. At determined timepoints (1, 2, 4, and 6 h) after incubation, a group of dishes (*n* = 3) was removed from the incubator. Media was then discarded, and cells were washed with 1 mL of serum-free media. Thereafter, cells were incubated with 0.5 mL urea–glycine buffer pH 2.5 (acid wash) for 5 min on ice. This acid wash was then collected. An additional 0.5 mL of the acid wash was also used to wash the cells, and this fraction was collected immediately. Cells were then incubated with 0.5 mL 1 M NaOH solution for at least 30 min at 37 °C to lysate the cells (base wash). Cells were additionally washed with 0.5 mL base wash. Both acid and base washes were measured for radioactivity using automated γ-spectrometer.

### 4.8. Biodistribution and In Vivo Targeting

The animal experiments were planned and performed in accordance with national legislation on laboratory animal protection. The animal studies were approved by the local ethics committee for animal research in Uppsala, Sweden (C85/15). 

Comparative biodistribution studies of ^99m^Tc-labeled (Z_HER2:2891_)_2_–ABD–MC-DM1, (Z_HER2:2891_)_2_–ABD–E_3_–MC-DM1, and (Z_HER2:2891_)_2_–ABD–E_6_–MC-DM1 were performed in female BALB/c nude mice (Scanbur A/S, Karlslunde, Denmark). Two weeks before the start of the experiment, 36 mice (6–8 weeks old) were injected with 10 × 10^6^ SKOV-3 cells/per mouse (HER2+) in the right hind leg. The mice (18.4 ± 1.4 g) were randomized to nine groups, with four mice in each group. Animals were injected intravenously with 6 μg (of each conjugate) per animal in 100 μL PBS containing 2% BSA. The injected radioactivity was calculated to give 30 kBq per mouse by the time of dissection. At predetermined timepoints (4, 24, and 46 h p.i.) mice were euthanized by overdosing of anesthesia (Ketalar (ketamine): 10 mg/mL, Pfizer AB, Sweden; Rompun (xylazine): 1 mg/mL, Bayer AG, Leverkusen, Germany) followed by heart puncture and exsanguination. Organs and tissue samples were collected and weighed, and the radioactivity was measured using an automated γ-spectrometer.

To demonstrate the specific delivery of ^99m^Tc-labeled (Z_HER2:2891_)_2_–ABD–MC-DM1, (Z_HER2:2891_)_2_–ABD–E_3_–MC-DM1, and (Z_HER2:2891_)_2_–ABD–E_6_–MC-DM1 to HER2-expressing tumors, an in vivo specificity study was performed. For this, an additional 12 BALB/c nude mice were xenografted with 5 × 10^6^ RAMOS (HER2) lymphoma cells in the right hind leg. Each group of four mice (*n* = 4) were i.v. injected with 6 µg (30 kBq) of the respective conjugate in 100 µL PBS containing 2% BSA. Mice were euthanized at 46 h p.i. and treated as mentioned above.

## 5. Conclusions

In conclusion, this study demonstrated that insertion of a polyglutamate spacer is an effective strategy to decrease hepatic uptake of affinity protein drug conjugates. The use of the hydrophilic and negatively charged glutamate spacer provided, by far, the lowest level of hepatic uptake for AffiDCs. Accumulation in other organs and tissues was also low, and no influence on the HER2-mediated tumor uptake was observed. We believe that the approach described here represents a means for the development of other targeting affinity protein drug conjugates for treatment of disseminated cancers and to facilitate their clinical translatability.

## Figures and Tables

**Table 1:** Biochemical characterization of the conjugates.

Conjugates	Purity (%) ^a^	Calc. Mw (Da)	Found Mw (Da) ^b^
(Z_HER2:2891_)_2_–ABD–MC-DM1	>95	21,006.3	21,005.8
(Z_HER2:2891_)_2_–ABD–E_3_–MC-DM1	>95	21,393.6	21,393.0
(Z_HER2:2891_)_2_–ABD–E_6_–MC-DM1	>95	21,781.0	21,780.1
(Z_HER2:2891_)_2_–ABD–IAA	>95	20,219.9	20,219.5

^a^ Determined by analytical RP-HPLC; ^b^ Mass spectrometry was used to determine the molecular weight (Mw) of the conjugates. Deconvolution was performed to determine the monoisotopic molecular weight of the proteins.

**Table 2:** Affinity constants for conjugates interacting with HER2.

Measurment	(Z_HER2:2891_)_2_–ABD–IAA	(Z_HER2:2891_)_2_–ABD–MC-DM1	(Z_HER2:2891_)_2_–ABD–E_3_–MC-DM1	(Z_HER2:2891_)_2_–ABD–E_6_–MC-DM1
k_a_ (1/M·s)	3.0 × 10^5^	7.9 × 10^4^	5.6 × 10^4^	5.5 × 10^4^
k_d_ (1/s)	9.6 × 10^−5^	1.4 × 10^−4^	1.3 × 10^−4^	1.5 × 10^−4^
K_D_ (M)	3.2 × 10^−10^	1.7 × 10^−9^	2.4 × 10^−9^	2.8 × 10^−9^

**Table 3:** Affinity constants for conjugates interacting with serum albumin.

Measurment	(Z_HER2:2891_)_2_–ABD–MC-DM1		(Z_HER2:2891_)_2_–ABD–E_3_–MC-DM1		(Z_HER2:2891_)_2_–ABD–E_6_–MC-DM1	
	HSA	MSA	HSA	MSA	HSA	MSA
k_a_ (1/M·s)	3.3 × 10^5^	6.0 × 10^5^	1.7 × 10^5^	2.8 × 10^5^	1.6 × 10^5^	3.0 × 10^5^
k_d_ (1/s)	1.9 × 10^−4^	1.5 × 10^−3^	2.0 × 10^−4^	2.0 × 10^−3^	1.9 × 10^−4^	2.4 × 10^−3^
K_D_ (M)	5.7 × 10^−10^	2.5 × 10^−9^	1.2 × 10^−9^	7.2×10^−9^	1.2 × 10^−9^	8.0 × 10^−9^

**Table 4:** In vitro cytotoxicity of the conjugates.

Cell Line	IC_50_ (nM)				
	(Z_HER2:2891_)_2_–ABD–MC-DM1	(Z_HER2:2891_)_2_–ABD–E_3_–MC-DM1	(Z_HER2:2891_)_2_–ABD–E_6_–MC-DM1	(Z_HER2:2891_)_2_–ABD–IAA	(Z_Taq_)_2_–ABD–MC-DM1
AU565	0.22	0.48	0.48	NM ^b^	NM
	(0.16–0.26) ^a^	(0.38–0.70)	(0.33–0.69)		
SKBR-3	0.14	0.17	0.38	NM	NM
	(0.10–0.19)	(0.13–0.23)	(0.27–0.43)		
SKOV-3	47.0	82.8	116	NM	350

^a^ Ranges in parenthesis correspond to 95% confidence interval; ^b^ Not measured.

**Table 5:** Labeling yield and radiochemical purity of ^99m^Tc-labeled AffiDCs.

Conjugate	Yield ^a^ (%)	Radiochemical Purity (%) ^b^	Stability Under Histidine Challenge (%)			
			Histidine 5000×		Control	
			4 h	24 h	4 h	24 h
^99m^Tc-(Z_HER2:2891_)_2_–ABD–MC-DM1	63 ± 15	99.5 ± 0.6	98.6 ± 0.2	98.4 ± 1.1	100 ± 0.2	98.8 ± 0.8
^99m^Tc-(Z_HER2:2891_)_2_–ABD–E_3_–MC-DM1	61 ± 14	99.7 ± 0.3	99.2 ± 0.4	98 ± 1	99.9 ± 0.1	99.5 ± 0.1
^99m^Tc-(Z_HER2:2891_)_2_–ABD–E_6_–MC-DM1	58 ± 16	99.8 ± 0.3	98.9 ± 0.1	97.3 ± 1.1	99.3 ± 0.3	99.1 ± 0.1

^a^ Yield is calculated as % of conjugate-bound radioactivity from total added radioactivity determined by iTLC; ^b^ Radiochemical purity is calculated as proportion of conjugate-bound radioactivity from total radioactivity after purification.

## Supplementary Materials

The following are available online at https://www.mdpi.com/2072-6694/11/8/1168/s1, Figure S1: Scheme 1: Structures of the ^99m^Tc(CO)_3_ chelated by HEHEHE tag, Figure S2: In vivo specificity.
