# Supplementary material for: Incorporation of a Hydrophilic Spacer Reduces Hepatic Uptake of HER2-Targeting Affibody–DM1 Drug Conjugates
Source: Cancers (Basel). 2019 Aug 14;11(8):1168. doi: 10.3390/cancers11081168 (PMC6721809; doi:10.3390/cancers11081168)
Supplement: Supplementary file 1 [file cancers-11-01168-s001.pdf]

Supplementary Material

Incorporation of a Hydrophilic Spacer Reduces Hepatic Uptake of HER2-Targeting Affibody-DM1 Drug Conjugates

Haozhong Ding, Mohamed Altai, Sara S. Rinne, Anzhelika Vorobyeva, Vladimir Tolmachev, Torbjörn Gräslund and Anna Orlova

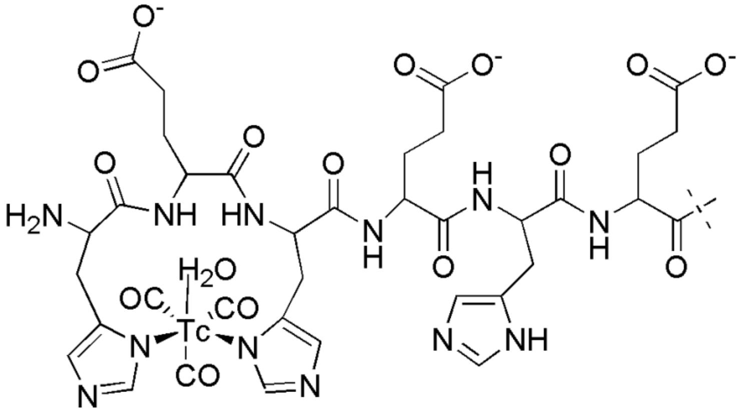

Figure S1. Scheme 1: Structures of the $^{99m}Tc(CO)_3$ chelated by HEHEHE tag.

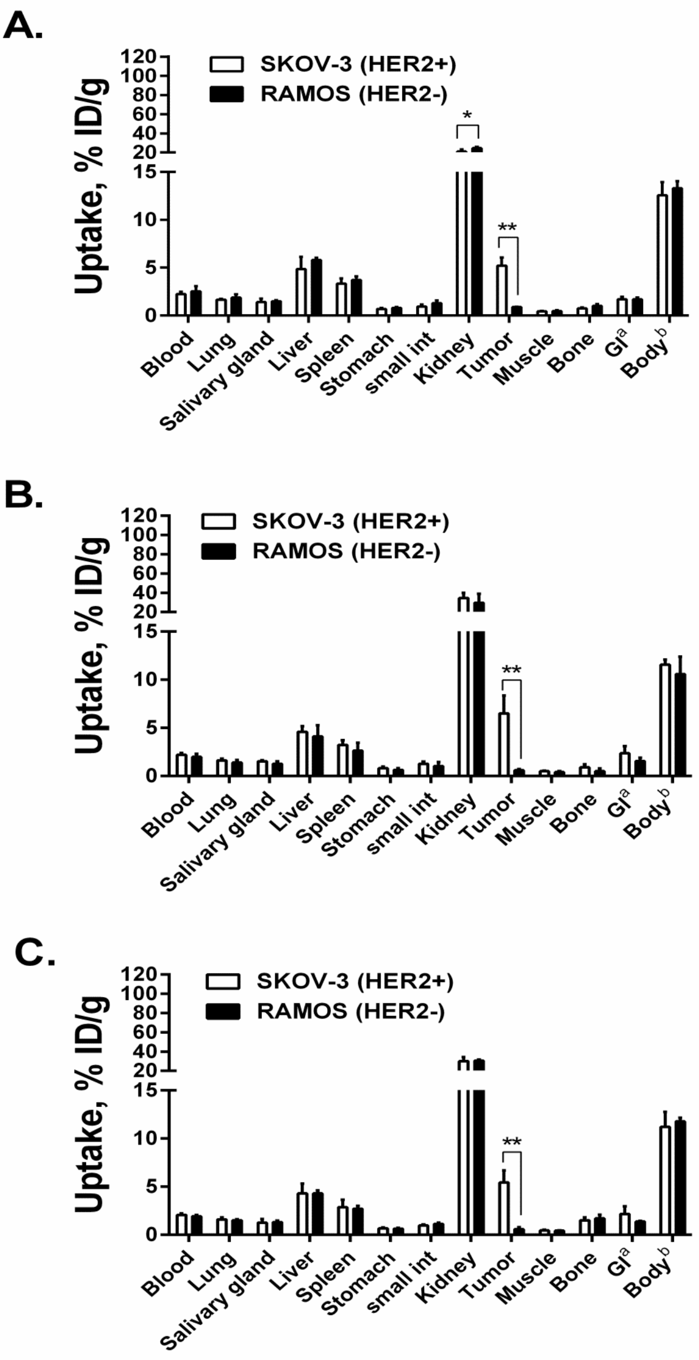

Figure S2. In vivo specificity. Comparative biodistribution of ^{99m}Tc–labeled $(Z_{HER2:2891})_2$-ABD-MC-DM1 (**A**), $(Z_{HER2:2891})_2$-ABD-E_3-MC-DM1 (**B**) and $(Z_{HER2:2891})_2$-ABD-E_6-MC-DM1 (**C**) expressed as %ID/g and presented as an average value from 4 animals ± 1 SD at 46 h after injection in mice bearing HER2+ SKOV-3 (empty) or HER2- RAMOS (filled) tumor xenografts. a,b Data are presented as %ID per whole sample. Data were assessed using student t-test test in order to determine significant differences ($p < 0.05$).

© 2019 by the authors. Licensee MDPI, Basel, Switzerland. This article is an open access article distributed under the terms and conditions of the Creative Commons Attribution (CC BY) license (http://creativecommons.org/licenses/by/4.0/).
